# Genetic Analysis of Antibody Response to Porcine Reproductive and Respiratory Syndrome Vaccination as an Indicator Trait for Reproductive Performance in Commercial Sows

**DOI:** 10.3389/fgene.2020.01011

**Published:** 2020-09-11

**Authors:** Leticia P. Sanglard, Rohan L. Fernando, Kent A. Gray, Daniel C. L. Linhares, Jack C. M. Dekkers, Megan C. Niederwerder, Nick V. L. Serão

**Affiliations:** ^1^Department of Animal Science, Iowa State University, Ames, IA, United States; ^2^Smithfield Premium Genetics, Rose Hill, NC, United States; ^3^Department of Veterinary Diagnostic and Production Animal Medicine, Iowa State University, Ames, IA, United States; ^4^Department of Diagnostic Medicine/Pathobiology, Kansas State University, Manhattan, KS, United States

**Keywords:** antibody response, porcine reproductive and respiratory syndrome vaccination, heritability, reproductive performance, genetic correlation, bivariate genome-wide association study

## Abstract

We proposed to investigate the genomic basis of antibody response to porcine reproductive and respiratory syndrome (PRRS) virus (PRRSV) vaccination and its relationship to reproductive performance in non-PRRSV-infected commercial sows. Nine hundred and six F1 replacement gilts (139 ± 17 days old) from two commercial farms were vaccinated with a commercial modified live PRRSV vaccine. Blood samples were collected about 52 days after vaccination to measure antibody response to PRRSV as sample-to-positive (S/P) ratio and for single-nucleotide polymorphism (SNP) genotyping. Reproductive performance was recorded for up to 807 sows for number born alive (NBA), number of piglets weaned, number born mummified (MUM), number of stillborn (NSB), and number of pre-weaning mortality (PWM) at parities (P) 1–3 and per sow per year (PSY). Fertility traits such as farrowing rate and age at first service were also analyzed. BayesC0 was used to estimate heritability and genetic correlations of S/P ratio with reproductive performance. Genome-wide association study (GWAS) and genomic prediction were performed using BayesB. The heritability estimate of S/P ratio was 0.34 ± 0.05. High genetic correlations (*r*_g_) of S/P ratio with farrowing performance were identified for NBA P1 (0.61), PWM P2 (-0.70), NSB P3 (-0.83), MUM P3 (-0.84), and NSB PSY (-0.90), indicating that genetic selection for increased S/P ratio would result in improved performance of these traits. A quantitative trait locus was identified on chromosome 7 (∼25 Mb), at the major histocompatibility complex (MHC) region, explaining ∼30% of the genetic variance for S/P ratio, mainly by SNPs ASGA0032113, H3GA0020505, and M1GA0009777. This same region was identified in the bivariate GWAS of S/P ratio and reproductive traits, with SNP H3GA0020505 explaining up to 10% (for NBA P1) of the genetic variance of reproductive performance. The heterozygote genotype at H3GA0020505 was associated with greater S/P ratio and NBA P1 (*P* = 0.06), and lower MUM P3 and NSB P3 (*P* = 0.07). Genomic prediction accuracy for S/P ratio was high when using all SNPs (0.67) and when using only those in the MHC region (0.59) and moderate to low when using all SNPs excluding those in the MHC region (0.39). These results suggest that there is great potential to use antibody response to PRRSV vaccination as an indicator trait to improve reproductive performance in commercial pigs.

## Introduction

Antibody response, measured as sample-to-positive (S/P) ratio, to porcine reproductive and respiratory syndrome (PRRS) in PRRS virus (PRRSV)-infected sows has been proposed as an indicator trait for selection for improved reproductive performance in sows during a PRRS outbreak (Serão et al., 2014). These authors reported that S/P ratio measured at approximately 46 days after the outbreak had high heritability (*h*^2^), with an estimate of 0.45. In addition, these authors showed that S/P ratio was highly genetically favorably correlated with litter size traits during PRRSV infection, such as number born alive (NBA), with an estimate of 0.73 and number of stillborn (NSB) of -0.72. Putz et al. (2019) also reported a high and negative *r*_g_ estimate between S/P ratio and NSB (-0.73). Nonetheless, these authors obtained a weak *r*_g_ estimate between S/P and NBA (0.05) and a low *h*^2^ estimate for S/P ratio (0.17). However, waiting for PRRS outbreaks to occur to collect data might limit the use of S/P ratio as a selection tool. Ideally, this strong relationship between S/P ratio and performance in PRRSV-infected sows would also be favorable in non-infected pigs.

A more feasible practice would be to use antibody response to PRRSV vaccination, which is a commonly used tool to control PRRS in commercial herds. PRRSV vaccination with a modified live virus (MLV) vaccine stimulates the same mechanisms of evasion as are developed during natural infection (Lopez and Osorio, 2004). Initially, T-cell-mediated response is stimulated with the production of interferon-gamma, and later non-neutralizing antibodies also play a role against the virus (Lopez and Osorio, 2004). Part of this response is controlled by genes located in the major histocompatibility complex (MHC), and this region may play a role in the relationship between immune response to vaccination and reproductive performance. Indeed, haplotypes in the MHC class I and II regions have been previously associated with reproductive traits, such as ovulation rate, embryo development, and litter size in non-infected pigs (Vaiman et al., 1998). Genetic variation in this region has been associated with S/P ratio in naturally PRRSV-infected sows (Serão et al., 2014) and in F1 replacement gilts (Serão et al., 2016). In addition, Serão et al. (2016) reported *h*^2^ estimates ranging from 0.28 to 0.47, as the proportion of seroconverted animals increased in the dataset. Although there was no confirmation on whether the replacement gilts in Serão et al. (2016) were PRRSV-vaccinated or naturally PRRSV-infected, or even both, these authors hypothesized that PRRSV vaccination would yield similar results at the genetic level for S/P ratio as in Serão et al. (2014). More directly investigating the relationship between antibody response to PRRSV vaccination and subsequent reproductive performance, Abella et al. (2019) evaluated the impact of PRRS vaccination on growing pigs at 6–7 weeks of age at the time of vaccination. These authors measured S/P ratio at 42 days after vaccination and obtained an *h*^2^ estimate of 0.69. Also, they developed a phenotyping criterion to discriminate susceptible and resilient sows based on viral load at 7 and 21 days post vaccination (Abella et al., 2019). At the phenotypic level, they reported that susceptible sows had greater antibody response to vaccination and higher NSB than those classified as resilient. However, in this study, they measured antibody in nursery pigs, when the energy being used for growth needs to be channeled for production of antibody after the vaccination, which may affect the relationship between antibody response and future performance.

Thus, in our study, we proposed to investigate the genomic basis of S/P ratio to PRRSV vaccination and its relationship with reproductive performance in non-infected commercial sows in an independent dataset (from a different breeding company) than the animals from Serão et al. (2016). For that purpose, we aimed to (1) estimate genetic parameters and genomic prediction accuracy (GPA) for antibody response to PRRSV vaccination, (2) assess the phenotypic and genetic relationships of antibody response to PRRSV vaccination with reproductive performance in commercial sows, and (3) identify regions of the genome [quantitative trait loci (QTLs)] that are associated with these traits.

## Materials and Methods

All methods described in this study were approved by the Institutional Animal Care and Use Committee at Iowa State University (IACUC# 6-17-8551-S).

### Phenotypic and Genotypic Data

Nine hundred and six naïve F1 (Landrace × Large White) replacement gilts from two commercial farms in North Carolina, United States, were vaccinated (139 ± 17 days old) intramuscularly with a commercial PRRS MLV vaccine (Ingelvac PRRS MLV, Boehringer Ingelheim Animal Health, Ames, IA, United States), following the manufacturer’s guidelines. Throughout the study, the farm performed periodic diagnostic tests for PRRSV and all animals (included or not in the study) did not show PCR-positive tests or signs of natural PRRSV infection. Blood samples were collected using Lavender Top Vacutainer tubes (Becton, Dickinson and Company, Franklin Lakes, NJ, United States) at approximately 50 days after vaccination in three contemporary groups (CGs), in which each CG represented 1 day of blood collection (52 and 53 days post vaccination for one farm, and 46 days post vaccination on the other farm). After collection, a drop of blood from each sample was used on blood cards (Neogen Genomics, Lincoln, NE, United States) for subsequent genotyping. The remaining blood samples were shipped to the Veterinary Diagnostic Laboratory at Iowa State University (Ames, IA, United States), where samples were processed using the laboratory’s standard procedures, for measurement of Immunoglobulin G against PRRSV, as S/P ratio, using a commercial ELISA test (IDEXX PRRS X3, IDEXX Laboratories Inc., Westbrook, ME, United States).

A subset of 807 animals from the animals with information of S/P ratio, located on PRRS-negative commercial farm, had farrowing performance recorded for up to three parities from January 2018 (∼150 days after blood collection) to December 2018 for the following litter size traits: NBA, number of stillborn (NSB), number born mummified (MUM), number of piglets weaned (NW), and number of pre-weaning mortality from the total number being weaned (PWM). Number born dead (NBD) was calculated as the sum of MUM and NSB, and total number born (TNB) was calculated as the sum of NBA and NBD. For animals with three parities, the number of piglets per sow per year (PSY) was calculated for each trait as the sum of the phenotype across the three parities divided by the difference in days between the third and first farrowing, multiplied by 365 days. We also analyzed fertility traits such as farrowing rate (FR), which was defined as the probability of an inseminated sow to farrow, age at first service (AFS), and farrowing interval (FI), which was the difference in days between the farrow date of parities 1 and 2, and between parities 2 and 3. The summary statistics of the data are presented in Table 1.

**TABLE 1:** Descriptive statistics of the data.

Traits	*N*	Mean	*SD*	Min	Max
S/P ratio	906	1.41	0.45	0.06	2.55
AFS	807	247.1	27.9	164.0	392.0
FI (P1 vs. P2)	616	161.8	28.5	129.0	343.0
FI (P2 vs. P3)	460	152.6	13.8	131.0	262.0
**Parity 1**					
FR	807	0.94	0.25	0.00	1.00
NBA	744	11.63	2.99	0.00	19.00
NSB	744	0.50	0.92	0.00	10.00
MUM	714	0.36	1.02	0.00	13.00
NBD	744	0.85	1.46	0.00	13.00
TNB	744	12.49	2.87	4.00	20.00
NW	735	11.03	2.47	0.00	15.00
PWM	735	2.27	2.07	0.00	11.00
**Parity 2**					
FR	755	0.81	0.40	0.00	1.00
NBA	608	12.67	3.35	0.00	22.00
NSB	608	0.45	0.86	0.00	7.00
MUM	608	0.28	0.88	0.00	12.00
NBD	608	0.73	1.31	0.00	12.00
TNB	608	13.40	3.37	4.00	24.00
NW	605	10.74	2.43	0.00	15.00
PWM	605	2.03	1.90	0.00	10.00
**Parity 3**					
FR	608	0.75	0.44	0.00	1.00
NBA	458	12.88	2.99	0.00	20.00
NSB	458	0.49	0.86	0.00	6.00
MUM	458	0.20	0.49	0.00	3.00
NBD	458	0.69	1.07	0.00	6.00
TNB	458	13.57	3.15	4.00	21.00
NW	458	9.01	4.08	0.00	15.00
PWM	458	2.05	2.03	0.00	12.00
**Per sow per year**					
NBA	448	44.45	7.43	21.00	66.36
NSB	448	0.63	0.56	0.00	2.36
MUM	448	0.41	0.53	0.00	2.90
NBD	448	1.00	0.70	0.00	3.06
TNB	448	47.15	7.61	21.00	73.48
NW	426	37.04	7.24	9.54	54.22
PWM	426	6.37	0.86	0.00	20.58

*AFS, age at first service (days); FI, farrow interval (days); P1, parity 1; P2, parity 2; P3, parity 3; FR, farrowing rate (1 if the sow farrowed or 0 if not after first service); NBA, number born alive; NSB, number of stillborn; MUM, number born mummified; NBD, number born dead; TNB, total number born; NW, number weaned; PWM, pre-weaning mortality; N, number of records.*

Blood cards were shipped to Neogen Genomics (Lincoln, NE, United States) for DNA extraction according to their standard procedures. Then, DNA from each animal was used for genotyping using the GGP Porcine HD (Neogen GeneSeek) for a total of 50,697 single-nucleotide polymorphisms (SNPs). Genotypes were set to missing if GC score < 0.50, SNPs with call rate < 0.90 were removed, and animals with genotype call rate < 0.90 were removed. After quality control, 45,536 SNPs and 906 animals were used for subsequent analyses. Positions of SNPs on the genome were based on the *Sus scrofa* 11.1 assembly.

### Statistical Analyses

#### Heritability and Genetic Correlations

Bayesian analysis (BayesC0; Habier et al., 2011) was used to estimate (co)variance parameters using the following model for each parity separately:

$$y=\mu +X\,b+W\,u+\sum_{i=1}^{k}z_{i}\,a_{i}\,\delta_{i}+e$$

where *y*_*ij*_ is a vector of phenotypic response (S/P ratio or reproductive performance); μ is the intercept; *X* is the incidence matrix relating the fixed effects to the response; ***b*** is a vector of fixed effects: CG for S/P ratio, farm for farrowing performance, and number of piglets cross-fostered and NBA as covariate (for NW and PWM); ***W*** is the incidence matrix relating the random effects to the response; ***u*** is the vector of random effects: combination of month/year of farrowing for NBA, NSB, MUM, NBD, and TNB, month/year of weaning for NW and PWM, and month/year of birth for AFS, PSY, FR, and FI traits; *z*_*i*_ is the vector of genotypes for SNP *i* (coded as 0, 1, and 2); α_*i*_ is the allele substitution effect of SNP *i*; δ_*i*_ is an indicator whether SNP *i* was included (δ_*i*_ = 1) or excluded (δ_*i*_ = 0) in the model for a given iteration of the Monte Carlo Markov chain (MCMC) (for BayesC0, δ_*i*_ = 1); and *e* is the vector of residuals. FR was a binary trait and was analyzed with a threshold model.

Bayesian analyses consisted of 50,000 MCMCs, with the first 5,000 discarded as burn-in. At every 100th iteration of the chain, the breeding value of each individual used in the analysis was calculated as the sum of its genotypes multiplied by the sampled marker effects. The variance of the sampled breeding values was used as the sampled additive genetic variance in that iteration. The sampled additive genetic variance was divided by the sampled phenotypic variance (sum of sampled additive and residual variances) at each iteration to obtain the sampled heritability (*h*^2^). Then, the estimate of *h*^2^ was calculated as the posterior mean of the sampled *h*^2^. The posterior standard deviation of the *h*^2^ samples was used as the standard error of the estimate.

Bivariate analyses were performed between S/P ratio and reproductive performance using the same fixed and random effects as used for the univariate analyses, fitting the following model (Cheng et al., 2018a):

$$y_{j}=\mu +X\,b+W\,u+\sum_{i=1}^{m}z_{i\,j}\,D_{i}\,\beta_{i}+e_{j}$$

where *y*_*j*_ is a vector of phenotypes of *t* (*t* = 2) traits for individual *j*; μ is a vector of overall means for *t* traits; ***X*** is $\left[\begin{array}{c} X_{1}\\ 0 \end{array}\begin{array}{c} 0\\ X_{2} \end{array}\right]$, where *X*_1_ and *X*_2_are the incidence matrices relating the fixed effects to the response for S/P ratio (*k* = 1) and reproductive performance (*k* = 2), respectively; ***b*** = $\left[\begin{array}{c} b_{1}\\ b_{2} \end{array}\right]$, where *b*_*1*_ and *b*_2_are the vectors of fixed effects for S/P ratio and reproductive performance, respectively; *W* is the incidence matrix relating the random effects to the response (reproductive traits); *u* is the vector of random effects (month/year of farrow); *z*_*ij*_ is the genotype covariate at locus *i* for individual *j* (coded as 0, 1, and 2); *m* is the number of genotyped loci; *D*_*i*_ is a diagonal matrix with elements diag(*D*_*i*_) = *δ*_*i*_ = (*δ*_*i*1_,*δ*_*i*2_), where *δ*_*ik*_ is an indicator variable indicating if the marker effect of locus *i* for trait *k* is zero or not and, in this case, there are 2^*t*^ = 4 combinations for *δ*_*i*_: (0, 0), (1, 0), (0, 1), and (1, 1), with (0, 0) representing the proportion of markers not being fitted in both traits and so on (note that for BayesC0, all markers are being fitted for both traits); *β*_*i*_ is the vector of marker effect for loci *β*_*i*_, where ∼ MVN (0, ***G***), where $G\,=\,\left[\begin{array}{cc} \sigma_{\beta }^{2}\,i_{1} & \sigma_{\beta }\,i_{1,2}\\ \sigma_{\beta }\,i_{1,2} & \sigma_{\beta }^{2}\,i_{2} \end{array}\right]$ and is assumed to have an inverse Wishart prior distribution, $W_{t}^{-1}\,\left(S_{\beta },\,v_{\beta }\right)$; and *e*_*j*_ is the vector of residuals of *t* traits for individual *j*, where *e*_*j*_ **∼** MVN (0, ***R***), where $R\,=\,\left[\begin{array}{cc} \sigma_{e_{1}}^{2} & \sigma_{e_{1,2}}\\ \sigma_{e_{1,2}} & \sigma_{e_{2}}^{2} \end{array}\right]$ and is assumed to have an inverse Wishart prior distribution, $W_{t}^{-1}\,\left(S_{e},\,v_{e}\right)$.

The genetic correlation (*r*_g_) was estimated as the posterior mean of the correlation between the sampled genomic breeding values for each animal for each trait at each iteration and its standard deviation across iterations as the standard error. The proportion of the covariance between S/P ratio and reproductive performance that was explained by a 1-Mb window was calculated as the covariance between sampled window breeding for each animal obtained based on the SNPs in the 1-Mb window divided by the covariance between sampled breeding values for each animal obtained based on all SNPs across the genome. All analyses were performed in the *JWAS* package (Cheng et al., 2018b), written in the Julia programing language (Bezanson et al., 2017).

#### Genome-Wide Association Studies

Univariate and bivariate GWASs were performed for S/P ratio and for S/P ratio with reproductive performance, respectively, using the same models as before. First, BayesCπ (Habier et al., 2011) was used to estimate the proportion of markers to be fitted in the model. Then, BayesB (Habier et al., 2011) was used with the estimated π to identify QTL within 1-Mb SNP windows that explained most of the genetic variance accounted for by the SNPs and that had a posterior probability of inclusion (PPI) greater than 0.7 (Garrick and Fernando, 2013). A bivariate GWAS was performed when the estimate of *r*_g_ between S/P ratio, and the reproductive trait analyzed was larger than 0.50 to investigate QTL associated with the genetic covariance between the two traits. All analyses were performed using the *JWAS* package. Candidate genes in the QTL regions were identified using Ensembl BioMart (Kinsella et al., 2011). Linkage disequilibrium (LD) between SNPs within QTL regions was estimated as *r*^2^ using Plink (Purcell et al., 2007) and plotted using Haploview (Barrett et al., 2005).

#### Effect of Major Single-Nucleotide Polymorphisms on Antibody Response and Reproductive Traits

Results from the bivariate GWAS were used to perform additional analyses to evaluate the impact of SNPs on traits evaluated in this study. For this, SNPs with PPI > 0.70 or that explained more than 1% of the genetic variance explained by markers (TGVM) for each trait were simultaneously fitted as categorical fixed effects in the model used for estimation of genetic parameters. Orthogonal contrasts were used to test the additive and dominance effects for each marker. Significant associations and tendency were considered at the significance levels of *P* ≤ 0.05 and *P* ≤ 0.10, respectively. Analyses were performed in ASReml v4.0 (Gilmour et al., 2015).

#### Genomic Prediction

Genomic prediction was performed for S/P ratio using the same statistical model as used for GWAS, using BayesB (π = 0.995) and BayesC0. We used BayesB based on our results and on the literature, which showed that S/P ratio has a major QTL accounting for ∼25% of the genetic variance. The BayesB method assumes different genetic variance per locus, allowing for the QTL to be well captured while reducing the effect of the small-effect QTL. Due to this major QTL for S/P ratio, we also analyzed the data removing SNPs located in this major QTL. With many more markers to be estimated and assumption equal variance, we chose to use BayesC0, as this method is more suitable under these circumstances. Nonetheless, this is the first study showing the genomic prediction for S/P ratio to vaccination, and thus, we investigated both methods to identify the one that would be more accurate.

A three-fold cross-validation was used for genomic prediction analyses. For this, data from two CGs were used for training, and the data from the remaining CG were used as validation. This was repeated until all CGs were used as validation. The number of individuals in each validation dataset was 378, 257, and 252 for CGs 1, 2, and 3, respectively. Marker effects were estimated (i.e., trained) based on the training population in three scenarios: (1) using the whole genome (ALL), using markers for the MHC QTL on SSC 7 for each training population (MHC), and using the rest of markers not included in the MHC scenario (REST). For the MHC scenario, a GWAS was performed for each training population to preselect the markers. The regions defined for each training population were SSC 7 25–26 Mb (29 SNPs) for two training populations (CGs 1 with 3, and CGs 2 with 3) and SSC 7 23–26 Mb (81 SNPs) for the other training population (CGs 1 with 2). GPA was calculated as follows:

$$\text{GPA}=\,\frac{\frac{\sum_{i\,=\,1}^{3}n_{i}\,r_{i\,\left(G\,E\,B\,V,\,y^{{}^{*}}\right)}}{\sum_{i\,=\,1}^{3}n_{i}}}{\sqrt{h^{2}}}$$

where *n*_*i*_ is the number of individuals in the *i*th validation dataset (*i* = 1, 2, 3), *r*_i(GEBV,  y^^*)_ is the correlation between the genomic estimated breeding values (GEBVs) and phenotypes adjusted for estimates of fixed-effects (*y*^∗^) for the *i*th validation dataset, and *h*^2^ is the heritability estimate using the whole dataset. All analyses were performed using the *JWAS* package.

## Results

### Genetic Parameters

Estimates of *h*^2^ are presented in Table 2. *h*^2^ estimates for S/P ratio and AFS were moderate, with 0.34 and 0.29, respectively, and higher than those for reproductive performance. This indicates that a faster genetic progress could be obtained for S/P ratio compared with reproductive performance. All other fertility and litter size traits showed low *h*^2^ (<0.20) and were similar across parities and sows per year. The highest and lowest *h*^2^ estimates for these traits were 0.16 (FR P3) and <0.001 (NW and PWM P1, NSB and NBD P2, and MUM and NBD PSY), respectively. In general, the average *h*^2^ for FR (0.16) across parities was higher than for litter size traits (0.05). As expected, fertility and litter size traits had low *h*^2^ estimates, indicating slow genetic progress for these traits.

**TABLE 2:** Estimates of heritability (*h*^2^) and correlations (genetic and phenotypic; *r*_*g*_ and *r*_*p*_, respectively) between antibody response [sample-to-positive (S/P) ratio] to porcine reproductive and respiratory syndrome virus vaccination and reproductive traits.

Traits	*h*^2^	*r*_*g*_	*r*_*p*_
S/P ratio	0.34(0.05)	−	−
AFS	0.29(0.05)	−0.25(0.13)	−0.05(0.03)
FI (P1 vs. P2)	0.07(0.03)	−0.16(0.25)	−0.02(0.04)
FI (P2 vs. P3)	< 0.001(0.01)	0.07(0.26)	0.07(0.04)
**Parity 1**			
FR	0.16(0.04)	−0.04(0.13)	−0.04(0.03)
NBA	0.06(0.02)	0.61(0.16)	0.02(0.03)
NSB	0.03(0.02)	−0.02(0.20)	−0.001(0.03)
MUM	0.01(0.005)	−0.17(0.23)	−0.002(0.03)
NBD	0.05(0.02)	−0.05(0.29)	0.02(0.03)
TNB	0.08(0.04)	0.30(0.19)	0.01(0.03)
NW	< 0.001(0.01)	−0.35(0.26)	−0.08(0.03)
PWM	< 0.001(0.01)	−0.14(0.27)	0.09(0.03)
**Parity 2**			
FR	0.15(0.03)	−0.05(0.15)	−0.06(0.03)
NBA	0.10(0.04)	−0.15(0.21)	−0.09(0.03)
NSB	< 0.001(0.01)	−0.03(0.19)	−0.06(0.04)
MUM	0.02(0.01)	0.19(0.33)	0.05(0.03)
NBD	< 0.001(0.01)	−0.17(0.18)	−0.007(0.04)
TNB	0.15(0.05)	−0.19(0.63)	−0.10(0.04)
NW	0.11(0.06)	0.01(0.21)	0.04(0.04)
PWM	0.07(0.07)	−0.70(0.10)	−0.02(0.03)
**Parity 3**			
FR	0.16(0.03)	0.08(0.14)	−0.005(0.03)
NBA	0.04(0.01)	0.02(0.20)	0.001(0.04)
NSB	0.001(0.0007)	−0.84(0.05)	−0.01(0.04)
MUM	0.003(0.003)	−0.83(0.11)	0.01(0.04)
NBD	0.01(0.009)	−0.19(0.23)	0.002(0.04)
TNB	0.12(0.03)	0.01(0.22)	0.006(0.04)
NW	0.08(0.09)	−0.11(0.38)	−0.04(0.04)
PWM	0.21(0.14)	−0.18(0.44)	−0.02(0.03)
**Per sow per year**			
NBA	0.07(0.04)	0.20(0.16)	−0.07(0.04)
NSB	0.01(0.005)	−0.90(0.05)	−0.07(0.04)
MUM	< 0.001(0.01)	−0.03(0.30)	0.01(0.04)
NBD	< 0.001(0.01)	−0.24(0.36)	−0.06(0.05)
TNB	0.09(0.05)	−0.02(0.26)	−0.08(0.04)
NW	0.04(0.09)	−0.17(0.37)	−0.06(0.04)
PWM	0.10(0.10)	−0.13(0.27)	0.06(0.04)

*AFS, age at first service; FI, farrow interval; P1, parity 1; P2, parity 2; P3, parity 3; FR, farrowing rate; NBA, number born alive; NSB, number of stillborn; MUM, number of mummies; NBD, number born dead (NSB + MUM); TNB, total number born (NBA + NBD); NW, number weaned; PWM, pre-weaning mortality.*

The *r*_g_ estimates between S/P ratio with reproductive traits are presented in Table 2. There were few moderate-to-high *r*_g_ estimates with favorable direction, such as between S/P ratio with NBA P1 (0.61), PWM P2 (-0.70), NSB P3 (-0.84), MUM P3 (-0.83), and NSB PSY (-0.90). These estimates indicate that selection for increased S/P ratio would result in improved farrowing performance for these traits. A moderate non-favorable *r*_g_ estimate was observed between S/P ratio and NW P1 (-0.35). For other traits, *r*_g_ estimates were overall low and favorable.

### Genome-Wide Association Studies

**FIGURE 1:**
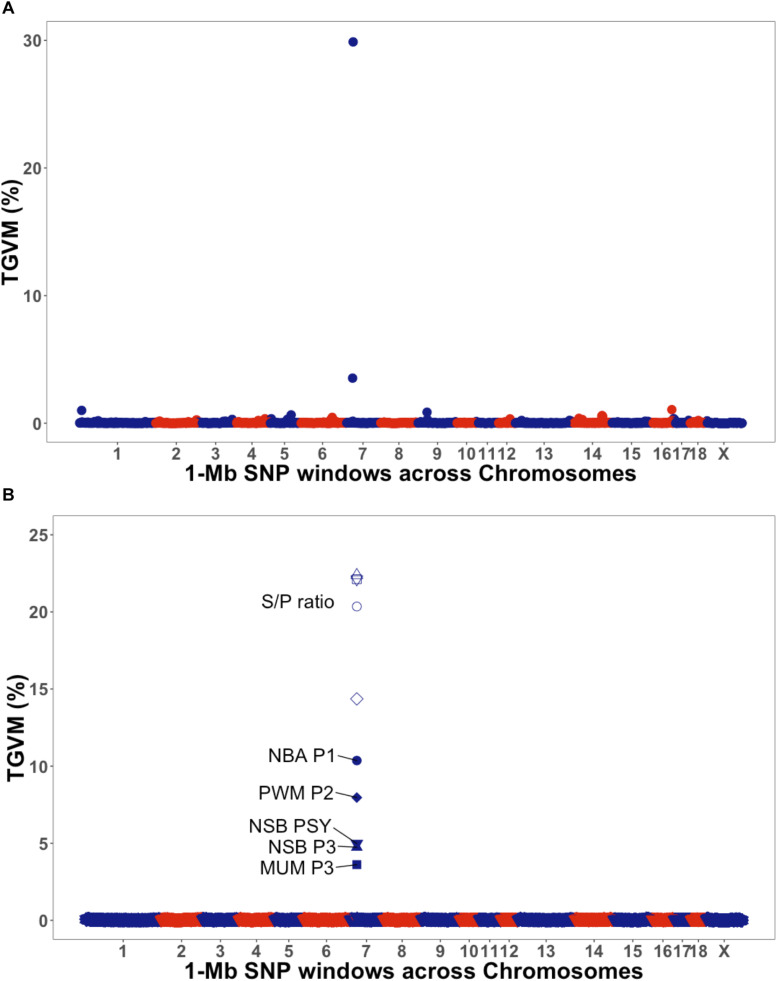
Manhattan plot for univariate and bivariate genome-wide association studies. The *y*-axis represents the percentage of total genetic variance explained for by markers (TGVM). The *x*-axis represents the position of 1-Mb single-nucleotide polymorphism (SNP) window across the genome. **(A)** Manhattan plot for univariate of antibody response [sample-to-positive (S/P) ratio] to porcine reproductive and respiratory syndrome virus vaccination (S/P ratio). **(B)** Manhattan plot for bivariate of S/P ratio (open symbols) and farrowing performance (solid symbols): number born alive at first parity (NBA P1; circle), pre-weaning mortality at second parity (PWM P2; diamond), number of stillborn at third parity (NSB P3; triangle), pre-weaning mortality at third parity (PWM P3; circle crossed), and number of piglets mummifies at third parity (NSB PSY; inverted triangle).

The GWAS results are presented in Figure 1 for the univariate and bivariate analyses. For the univariate analysis of S/P ratio (Table 3), we identified a region on *Sus scrofa* chromosome (SSC) 7 (23–26 Mb), the MHC region, explaining 30% of the TGVM (PPI = 1). This variance was mainly explained by SNPs ASGA0032113, H3GA0020505, and M1GA0009777, which explained 21% (PPI = 1), 10.5% (PPI = 0.93), and 3.5% (PPI = 0.78) of the TGVM, respectively.

**TABLE 3:** Results for the genome-wide association study (GWAS) analysis for sample-to-positive (S/P) ratio (single trait) to porcine reproductive and respiratory syndrome virus vaccination measure as and bi-trait for S/P ratio and reproductive performance.

Traits	SSC	Window		#SNPs	S/P ratio		Reproductive trait	
					
		Start (Mb)	End (Mb)		TGVM (%)	PPI	TGVM (%)	PPI
*Single trait*	7	25,003,013	25,967,157	29	30.00	1.00	–	–
	7	23,037,875	23,985,825	18	3.50	0.73	–	–
*Bi-trait*								
NBA P1	7	25,003,013	25,967,157	29	20.35	0.99	10.36	0.84
PWM P2	7	25,003,013	25,967,157	29	19.67	0.84	7.96	0.76
NSB P3	7	25,003,013	25,967,157	29	22.38	1.00	4.74	0.42
MUM P3	7	25,003,013	25,967,157	29	22.12	0.99	3.61	0.58
NSB PSY	7	25,003,013	25,967,157	29	21.80	0.99	4.98	0.51

*NBA, number born alive; PWM, pre-weaning mortality; NSB, number of stillborn; MUM, number of piglets mummified; P1, parity 1; P2, parity 2; and P3, parity 3; PSY, pigs per sow per year; SSC, *Sus scrofa* chromosome; #SNPs, number of SNPs within the window; TGVM, total genetic variance explained by the markers in the window; PPI, posterior probability of inclusion.*

For the bivariate analysis between S/P ratio and reproductive traits showing *r*_g_ > 0.50 with S/P ratio (Table 2), results are presented in Table 3. For all analyses, a similar region identified for the univariate analysis of S/P ratio on SSC 7 was found for all the traits, on the MHC class II region (SSC 7, 25–26 Mb; Figure 1B). This region explained 10.4% (PPI = 0.84), 7.9% (PPI = 0.53), 4.7% (PPI = 0.42), 3.6% (PPI = 0.58), and 4.9% (PPI = 0.51) of the TGVM for NBA P1, PWM P2, NSB P3, MUM P3, and NSB PSY, respectively; and an average of 21.4% (*SD* = 1.1%; PPI ≥ 0.99) for S/P ratio across all bivariate GWASs. Additionally, this region explained 34, 25, 26, 35, and 90% of the genetic covariance explained for by the markers (TGCoVM) between S/P ratio with NBA P1, PWM P2, NSB P3, MUM P3, and NSB PSY, respectively. These results indicate that this major region on the MHC for S/P ratio is also associated with farrowing traits.

**FIGURE 2:**
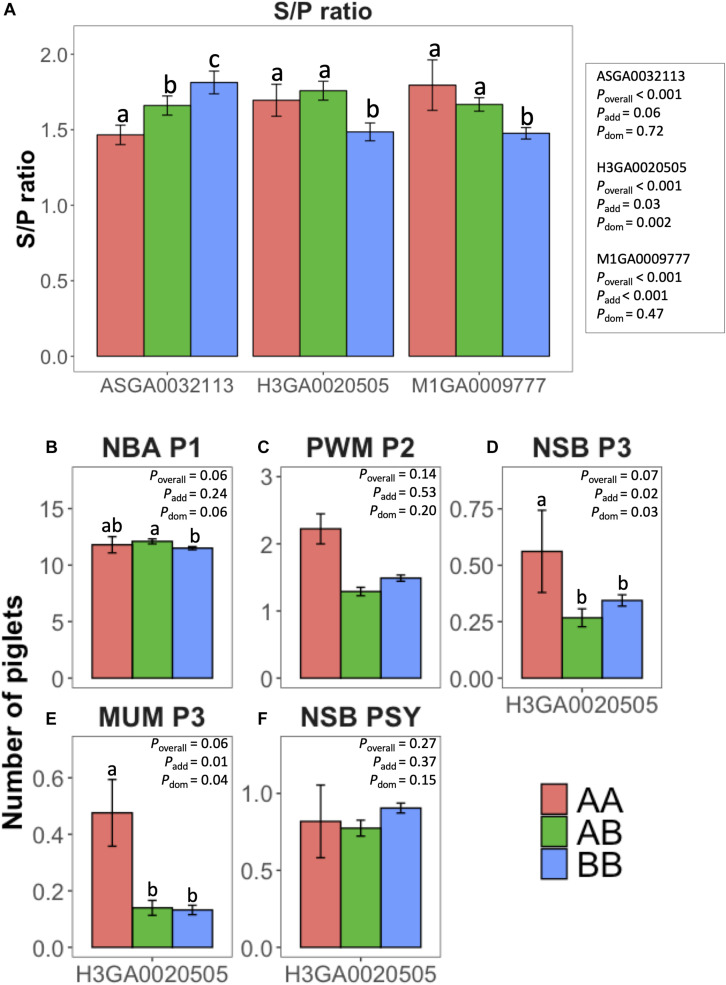
Effect of selected single-nucleotide polymorphisms (SNPs) from univariate [antibody response (S/P ratio) to porcine reproductive and respiratory syndrome virus vaccination] and bivariate (farrowing performance) genome-wide association studies. *y*-axis represents least squares means for **(A)** S/P ratio, **(B)** number born alive at first parity (NBA P1), **(C)** pre-weaning mortality at second parity (PWM P2), **(D)** number of stillborn (NSB) at third parity (NSB P3), **(E)** number of piglets mummified at third parity (MUM P3), and **(F)** NSB per sow per year (NSB PSY). Colors represent the genotypes AA (coral), AB (green), and BB (blue) of the SNP evaluated: ASGA0032113, M1GA0009777, and H3GA0020505. Error bars represent standard errors. Different letters over the bars represent significant difference between the genotypes within a SNP. *P*-values for the overall *F*-test, additive effect, and dominance effect of each SNP are represented by *P*_*overall*_, *P*_*add*_, and *P*_*dom*_, respectively.

The effect of the main SNPs explaining most of the %TGVM in the QTL identified in the univariate (S/P ratio; ASGA0032113, H3GA0020505, and M1GA0009777) and bivariate analysis (reproductive traits; H3GA0020505) is shown in Figure 2. These three SNPs were significantly (*P* < 0.001) associated with S/P ratio to PRRSV vaccination. The additive effect was significant (*P* ≤ 0.06) for all of them, and the dominance effect was significant for H3GA0020505 (*P* = 0.002). For H3GA0020505 and M1GA0009777, genotypes AA (1.7 ± 0.11 and 1.8 ± 0.17, respectively) and AB (1.8 ± 0.06 and 1.7 ± 0.04, respectively) had greater S/P ratio than BB (1.5 ± 0.06 and 1.5 ± 0.04, respectively), while for ASGA0032113, S/P ratio increased from AA to BB, with 1.5 ± 0.06, 1.7 ± 0.06, and 1.8 ± 0.08, for AA, AB, and BB, respectively (Figure 2A).

The main SNP associated with farrowing performance in the bivariate analysis was H3GA0020505. This marker had significant (*P* ≤ 0.07) effect on NBA P1, MUM P3, and NSB P3. For these traits, there was a dominance effect (*P* ≤ 0.06), whereas there was an additive effect (*P* = 0.01) also for MUM P3 (Figure 2B). For NBA, AB (12.1 ± 0.22) had greater NBA than BB (11.5 ± 0.14), but these two genotypes did not differ from AA (11.8 ± 0.72). For MUM P3, AA (0.06 ± 0.02) had worse performance than AB (0.03 ± 0.001) and BB (0.03 ± 0.001).

### Effect of Major Histocompatibility Complex Single-Nucleotide Polymorphisms on Antibody Response and Reproductive Traits

The effect of the main SNPs explaining most of the %TGVM in the QTL identified in the univariate (S/P ratio; ASGA0032113, H3GA0020505, and M1GA0009777) and bivariate analyses (reproductive traits; H3GA0020505) is shown in Figure 2. These three SNPs were significantly (*P* < 0.001) associated with S/P ratio to PRRSV vaccination. There was a tendency (*P* ≤ 0.06) for the additive effect for all of them, and the dominance effect was significant for H3GA0020505 (*P* = 0.002). For H3GA0020505 and M1GA0009777, genotypes AA (1.7 ± 0.11 and 1.8 ± 0.17, respectively) and AB (1.8 ± 0.06 and 1.7 ± 0.04, respectively) had greater S/P ratio than BB (1.5 ± 0.06 and 1.5 ± 0.04, respectively), while for ASGA0032113, S/P ratio increased from AA to BB, with 1.5 ± 0.06, 1.7 ± 0.06, and 1.8 ± 0.08, for AA, AB, and BB, respectively (Figure 2A).

The main SNP associated with farrowing performance in the bivariate analysis was H3GA0020505. This marker shows a tendency (*P* ≤ 0.07) for NBA P1, MUM P3, and NSB P3. For NBA P1, there was a tendency for the dominance effect (*P* = 0.06), whereas for MUM P3 and NSB P3, the additive and dominance effects were significant (*P* ≤ 0.04; Figure 2B). For NBA, AB (12.1 ± 0.22) had greater NBA than BB (11.5 ± 0.14), but these two genotypes did not differ from AA (11.8 ± 0.72). For MUM P3, AA (0.06 ± 0.02) had worse performance than AB (0.03 ± 0.001) and BB (0.03 ± 0.001).

### Genomic Prediction

**FIGURE 3:**
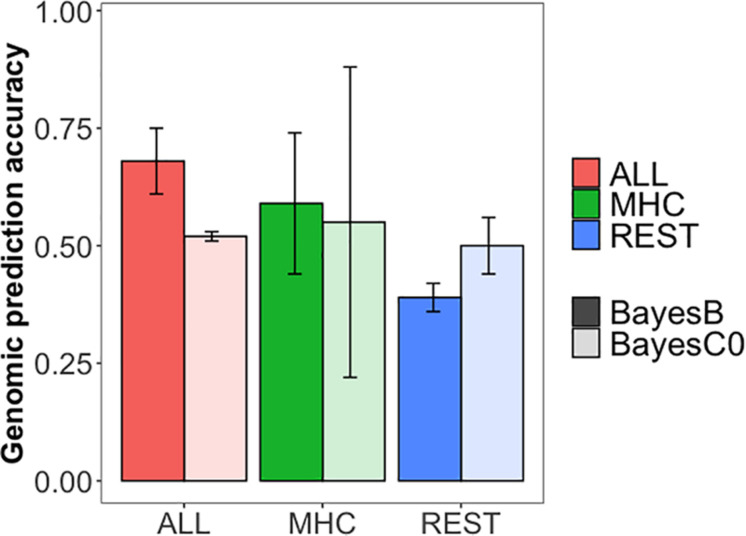
Genomic prediction accuracies for antibody response [sample-to-positive (S/P) ratio] to porcine reproductive and respiratory syndrome virus vaccination. The colors indicate the set of single-nucleotide polymorphisms (SNPs) used in the analysis: whole dataset (ALL, coral), only the SNPs in the major histocompatibility complex region (chromosome 7, 23, to 26 Mb; MHC, green), and only SNPs outside the MHC region (REST, blue). The transparency indicates the method of estimation used: darker colors represent BayesB analysis (Pi = 0.995), and lighter colors represent BayesC0. Error bars represent the standard deviation across three cross-validation folds.

GPAs for S/P ratio are presented in Figure 3 for three scenarios: (1) using all the SNPs (ALL), (2) using SNPs on the MHC region, and (3) using SNPs only outside the MHC region (REST). The MHC region defined for the training populations without CG 1 or 2 was the same between them and narrower than the region defined by training population without CG 3. The TGVM explained by the MHC region for region SSC 7 25–26 Mb was 26.06% (PPI = 1.00; CGs 2 and 3 in the training dataset), 23.94% (PPI = 1.00; CGs 1 and 3 in the training dataset), and 30.22% (PPI = 1; CGs 1 and 2 in the training dataset), whereas for the region SSC 7, 23–26 Mb was 10.0% (PPI = 0.95; CGs 1 and 2 in the training dataset).

GPAs were moderate to high, ranging from 0.39 for REST (BayesB) to 0.59 for ALL (BayesB). GPAs were higher for BayesB than for BayesC0 for scenarios ALL and MHC, but not for REST. This may be explained by the major QTL on the MHC region identified for all training population and was excluded in the scenario REST. We observed a large standard deviation for the MHC scenario when using BayesC0, much larger than when using BayesB. The GPAs for training population without CGs 1, 2, and 3 were 0.71, 0.68, and 0.17, respectively, for BayesC0, and were 0.58, 0.68, and 0.51, respectively, for BayesB. Thus, selection and training of markers using CGs 1 and 2 and validating CG 3 did not work well using BayesC0. Among scenarios, ALL showed better accuracy than did MHC and REST for BayesB, whereas ALL and MHC were comparable for BayesC0. Although there was substantial decrease in GPA from MHC to REST for BayesB, this was not the case for BayesC0. In fact, overall, BayesC0 had similar GPAs across all scenarios.

## Discussion

### Genetic Parameters

#### Heritabilities

The *h*^2^ estimates reported for litter size traits in this study were slightly lower than estimates from literature using commercial F1 gilts (Ehlers et al., 2005; Strange et al., 2013). Nonetheless, these were low as expected, and within those previously reported in the literature when accounting for the SE of the estimates. The *h*^2^ estimate for AFS was moderate but higher than previously shown in F1 (Large White × Yorkshire) gilts (0.16; Kumari and Rao, 2010). Our estimate was more similar to what has been reported for Landrace (0.31; Holm et al., 2005). The *h*^2^ estimates for PSY traits were also low. Noppibool et al. (2017) reported *h*^2^ of 0.07 ± 0.03 for NBA PSY and 0.13 ± 0.03 for NW PSY in purebred sows (Landrace and Yorkshire). Abell et al. (2013) reported an *h*^2^ of 0.11 ± 0.01 in a study including Landrace, Large White, and F1 sows. To the best of our knowledge, there are no reports in the literature regarding the mortality traits PSY. In our study, the *h*^2^ for FR was low but slightly higher to what has been reported by Bloemhof et al. (2015) of 0.10 ± 0.01 in a crossbred (Yorkshire × Landrace) population. For FI, the estimates of heritability in the literature are low (<0.10) in pure (Cavalcante Neto et al., 2009) and crossbred pig populations (Nagyné-kiszlinger et al., 2013), similar to what we have found. These results reaffirm that selection over litter size traits is challenging due to its low heritability.

In this study, F1 replacement gilts followed the standard acclimation procedures for the farms enrolled in our study. Moreover, these animals were sourced from high health multiplier herds and represent standard high-producing sows in the US swine industry. Therefore, our results are well representative of current genetics used for commercial production.

The number of studies investigating the genetic variation for antibody response to PRRSV has increased in the literature in the past few years. Studies vary on the age of animals (young and adult), type of exposure to PRRSV (vaccination, infection or both), and the assay used to measure antibodies. We measured S/P ratio ∼52 days after vaccination using a commercial ELISA test, when animals were ∼34 weeks old, and we obtained a moderate *h*^2^ estimate for S/P ratio, with 0.34 ± 0.05. The first *h*^2^ estimate for S/P ratio reported in the literature was by Serão et al. (2014), with 0.45 ± 0.13 in multiparous purebred sows during a PRRS outbreak. These authors measured S/P ratio at about 46 days after the PRRS outbreak using the same ELISA test utilized in our study. Also using purebred pregnant sows under a PRRS outbreak, Putz et al. (2019) reported a much lower *h*^2^ estimate, of 0.17 ± 0.05 during the PRRS outbreak. In their study, antibody response to PRRSV was measured at about 60 days after the PRRS outbreak. There were three main differences between Serão et al. (2014) and Putz et al. (2019) that could be associated with different estimates of *h*^2^ observed. First, the time of sample collection in Putz et al. (2019) was of about 2 weeks after the PRRS outbreak than in Serão et al. (2014), which could indicate a different immunological response of the animals (further discussion below). Second, Putz et al. (2019) used a different method of antibody measurement, based on microsphere (or microbead) assay, in contrast to using a standard commercial ELISA test. Finally, all animals in Putz et al. (2019) were re-exposed to the PRRSV before (∼30 days) serum samples were collected to measure antibody. However, Hickmann et al. (2019) reported moderate *h*^2^ estimates of S/P in Duroc (0.33 ± 0.06) and Landrace (0.28 ± 0.07) sow populations during a PRRS outbreak, measured about 54 days after the outbreak using the same ELISA test used in our study. As in Putz et al. (2019), these animals were re-exposed to the PRRSV prior to collection of samples to measure antibody response. Therefore, low *h*^2^ estimate found in Putz et al. (2019) for S/P ratio is likely associated with the differences in diagnostic assays.

Differently than in the studies described above, in our study, we investigated the relationship between S/P ratio and vaccination with reproductive performance in non-infected sows. Serão et al. (2016), using the same PRRS ELISA test as in our study, reported *h*^2^ estimates ranging from 0.28 ± 0.04 to 0.47 ± 0.06, as the proportion of seroconverted animals increased in the dataset; however, there was no confirmation on whether the replacement gilts in were PRRSV-vaccinated or PRRSV-infected or even both. More similar to our study, Abella et al. (2019) estimated *h*^2^ for S/P ratio at 42 days after PRRSV vaccination and obtained a higher *h*^2^ estimate than we observed in our study (0.69 ± 0.10). Although they used the same ELISA test as us, the animals in their study were growing pigs at 6–7 weeks of age, and it is possible that the redirect of energy being used to growth for antibody production may have affected future performance. Another important point to be highlighted is that in the study by Abella et al. (2019), the herds were endemic for PRRS, and, therefore, the animals were in constant health challenge during vaccination and farrowing. Therefore, our study is the first one, to the best of our knowledge, to estimate *h*^2^ for S/P ratio in PRRS-vaccinated F1 replacement gilts.

Several environmental factors may be involved in the different *h*^2^ estimates observed across the different studies, such as the time of sample collection, the assay used for antibody measurement, the strains affecting the population, breeds, and genetic background, among others. The difference observed between our studies and PRRS outbreak studies may be the higher viral load during an outbreak compared with vaccination, although the use of MLV stimulates a similar immune response when compared with natural infection (Ellingson et al., 2010). This may result in a stronger immune response, which exacerbates the genetic variability between the individuals. The humoral response to PRRSV is well known to be delayed (Montaner-Tarbes et al., 2019). Therefore, measuring antibody response of about 6–8 weeks after infection, as used in all these studies, seems reasonable to capture the genetic variability for antibody response to PRRSV (Lopez and Osorio, 2004).

Although slightly lower than expected, the *h*^2^ estimate for S/P ratio in this study was still substantially high, indicating that genetic selection to change response to PRRSV vaccine is possible. Nonetheless, assessment of *h*^2^ at different time points after PRRSV vaccination is needed to identify how S/P ratio response changes genetically across time.

#### Genetic Correlations of Sample-to-Positive Ratio With Reproductive Traits

In this study, we evaluated the relationship of S/P ratio in PRRSV-vaccinated gilts with their subsequent reproductive performance up to three parities. In general, results depended on the parity being analyzed. Phenotypic correlations were consistently close to 0, indicating that relationships between S/P ratio and performance may exist at the genetic and environmental levels depending on the parity and trait analyzed.

In our study, however, S/P ratio had high and moderate positive genetic correlations with NBA P1 (0.60) and TNB P1 (0.30), respectively. The low negative *r*_g_ between S/P ratio with MUM P1 (-0.17) and NSB P1 (-0.05) may explain the lower *r*_g_ between S/P ratio with TNB P1 compared with the *r*_g_ with NBA P1, which indicates that selection for increased S/P ratio would increase NBA and decrease mortality traits at parity 1. Also, S/P ratio was highly negatively correlated with MUM P3 (-0.83) and NSB P3 (-0.84), indicating that the use of S/P ratio as a selection tool would have positive long-term effects. For the *r*_g_ estimates between S/P ratio with NSB P3 (-0.84) and MUM P3 (-0.83), although the standard errors for these estimates were low, these estimates were inconsistent with the one for NBD (-0.19), since NBD is calculated as a function of NSB and MUM. The *h*^2^ estimates for NSB P3 (0.001) and MUM P3 (0.003) were very low in our study and, thus, have very low genetic variances for these traits in comparison with their respective phenotypic variances. Therefore, although highly genetically correlated with S/P ratio, the very low *h*^2^ estimates for these traits indicate that there is limited genetic improvement of these traits. On the other hand, the posterior probabilities of these *r*_g_ estimates being smaller than zero were of 1 (data not shown), indicating that there is a negative genetic correlation between S/P ratio with NSB P3 and MUM P3.

Although not directly comparable, our *r*_g_ estimates of S/P ratio with NBA and NSB are similar to those reported by Serão et al. (2014), at 0.73 and -0.72, respectively, suggesting that the proposition of using S/P ratio as an indicator trait for reproductive performance in PRRSV-infected sows should be more general, as this relationship seems to also hold between S/P ratio in vaccinated gilts and reproductive performance in the absence of PRRSV infection. Putz et al. (2019) also reported a similar estimate of *r*_g_ between S/P with NSB, of -0.73 during a PRRS outbreak but not for NBA, which was positive but very low (0.05). Abella et al. (2019) also investigated the relationship between S/P ratio in vaccinated young gilts and subsequent reproductive performance in endemic herds. In contrast to our results, they found a small but positive phenotypic correlation between antibody response to PRRSV vaccination and NSB when looking at extreme phenotypes for response to PRRSV vaccination (resistant and susceptible gilts). Nonetheless, it is important to highlight that, among other differences between the two studies, their study was based on a phenotypic relationship and not genetic. We also tried to investigate the extreme phenotypes based on S/P ratio and associate them with reproductive performance, but it was not significant for any of the traits investigated (data not shown).

In addition to these results, we also investigated the relationship of S/P ratio with AFS, FR, and PSY, which had not yet been reported in the literature. The *r*_g_ between S/P ratio and AFS was moderate and negative (-0.25) while null with FR, indicating that selection for increased S/P ratio would result in younger gilts being serviced. Analysis of PSY traits has the advantage of taking in consideration the interval between farrow events, which also reflects the overall farrowing efficiency of the sows. In general, the *r*_g_ estimates between S/P ratio with PSY trait were low to moderate, with exception of NSB, which was high and negative (-0.90), in accordance with the *r*_g_ estimate between S/P ratio and NSB P3 (-0.84).

**FIGURE 4:**
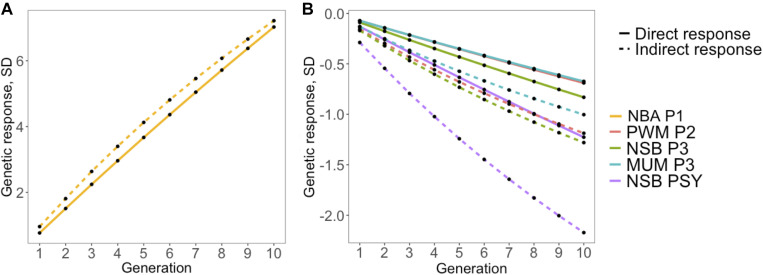
Simulated response to selection for reproductive performance after 10 generations based (indirect) or not (direct) on antibody response, measured as sample-to-positive (S/P) ratio, to porcine reproductive and respiratory syndrome virus vaccination. The *y*- and *x*-axes represent response to selection in genetic standard deviations and generations, respectively. Direct and indirect response to selection is represented by solid and dashed lines, respectively, assuming 5% selection intensity. Colors represent **(A)** number born alive at first parity at first parity (NBA P1; golden), **(B)** pre-weaning mortality at second parity (PWM P2; coral), number of stillborn (NSB) at third parity (NSB P3; green), number of piglets mummified at third parity (MUM P3; blue), and NSB per sow per year (NSB PSY; purple).

In general, results in our study indicate that genetic selection for increased antibody response to PRRSV vaccination would indirectly increase the reproductive performance in commercial sows. In order to evaluate the impact of direct selection for increased S/P ratio, we calculated the indirect response to selection on reproductive performance using the genetic parameters estimated in this study. For all deterministic simulations, intensity of selection was set to 5% for traits with *r*_g_ > 0.50 with S/P ratio (Figure 4). The correlated response to selection shown to be more efficient than direct response to those traits, with about 8% for NBA P1 (Figure 4A) and 72, 53, 49, and 77% more efficient for PWM P2, NSB P3, MUM P3, and NSB PSY (Figure 4B). Thus, selection for increased S/P ratio would result in substantial favorable gains for several reproductive traits. It is important to note that these calculations of efficiency of indirect response to selection when selecting for S/P ratio are simplistic, as in reality, animals are selected using an index with different economic weights. Therefore, a more comprehensive simulation is needed in follow up studies including costs associated with vaccination, measurements of S/P ratio, and genotyping. Nonetheless, the use of genomic selection allows breeding companies to use data on antibody response collected at the commercial levels to estimate breeding values for sires in the breeding herd with better antibody response to PRRS vaccination, which makes the proposed use of S/P ratio as an indicator trait for reproductive performance a feasible strategy.

### Genome-Wide Association Studies

The major QTL identified in the MHC region explaining 30% of the TGVM for S/P ratio in our study is the same previously associated with S/P ratio in sows naturally infected with wild-type PRRSV by Serão et al. (2014) and validated by Serão et al. (2016) in an independent dataset in F1 replacement gilts and by Hickmann et al. (2019) in two outbreak herds. Haplotypes in this region have also been previously significantly associated with production traits, such as average daily gain and backfat thickness in non-infected pigs (Jung et al., 1989; Vaiman et al., 1998). The MHC region spans from around 23–26 Mb in the SSC 7 (Hammer et al., 2020). Two of the main SNPs associated with S/P ratio, ASGA0032113 and H3GA0020505, were located at 25 Mb (MHC class II), while M1GA0009777 was located at 24 Mb (MHC class III).

The MHC region is well known for the presence of several immune-related genes with potential to be candidate causal genes associated with Ab response to PRRSV vaccination. The MHC class II harbors genes relating to peptide presentation of the adaptive immune system, such as swine leukocyte antigen (*SLA*)*-DR*, *SLA-DQ*, *SLA-DM*, and *SLA-DO* proteins; transporter-associated with antigen processing genes, such as ATP binding cassette subfamily B member transporter 1 (*TAP1*) and 2 (*TAP2*); and proteasomes, such as Proteasome 20S subunit beta 8 (*PSMB8*) and 9 (*PSMB9*) (Hammer et al., 2020). Interestingly, *SLA-DRA* has been shown to affect production and immune traits in swine (Lunney et al., 2009). In addition, Jiang et al. (2013) compared the lung transcriptome of PRRSV-infected gilts (at 6–8 weeks of age) and non-infected gilts and observed that *SLA-DRA*, *SLA-DRB1*, *SLA-DQA1*, *SLA-DQA2*, *SLA-DMB*, and *SLA-DOA* had significantly lower expression in the infected group compared with the non-infected group. *SLA-DRA*, *SLA-DRB1*, and *SLA-DQA1* are antigen binding genes, while *SLA-DMB* and *SLA-DOA* are involved in epitope loading of MHC class II molecules. Thus, all these genes are strong potential candidate genes since their lower expression may contribute to the attenuation of the immune response by the virus, which prolongs the PRRSV infection.

Similarly, the MHC class III region includes important genes for immune defense mechanisms and inflammation, including the tumor necrosis factor gene families, such as the tumor necrosis factor (*TNF*), lymphotoxin alpha (*LTA*), and lymphotoxin beta (*LTB*), components of the complement cascade, such as complement C2 (*C2*), complement C4A (*C4A*), and complement factor beta (*CFB*); heat shock proteins, such as *HSP1A*, *HSP1B*, and *HSP1L*; and genes with complex functions, such as tenascin XB (*TNXB*) and notch receptor 4 (*NOTCH4*). Some of them are potential candidate causal genes given their previous association with PRRSV. Among them, *C2* has been associated with PRRSV susceptibility and was downregulated in pregnant sows with higher antibody response at 35 days post PRRSV vaccination (Yang et al., 2018). In addition, *TNF* and heat shock proteins had significantly lower expression in the PRRSV-infected group compared with the non-infected group (Jiang et al., 2013). *C2* and *C4A* are involved in the activation of non-specific immune response (Vaiman et al., 1998), and their activation is associated with the mode of action of non-neutralizing antibody during PRRSV infection (Montaner-Tarbes et al., 2019). Heat shock proteins interact with viral protein and enhance the development of innate and adaptative system (Jiang et al., 2013). Therefore, the low expression of such genes may be associated with the weakened immune response observed during PRRSV infection.

Therefore, several genes located in this region are very likely to be associated with the strong relationship between the humoral immune response to PRRSV exposure (vaccination or natural infection) and the MHC region. However, in our study, we used SNP data to perform associations between them and variation in S/P ratio. Although we obtain strong and somewhat narrow associations, including the identification of specific SNPs explaining the majority of the variation, our analyses do not allow us to make any sort of cause and effect. Therefore, additional studies are needed to further investigate the causal variants that could be explained by the SNPs identified in our studies.

**FIGURE 5:**
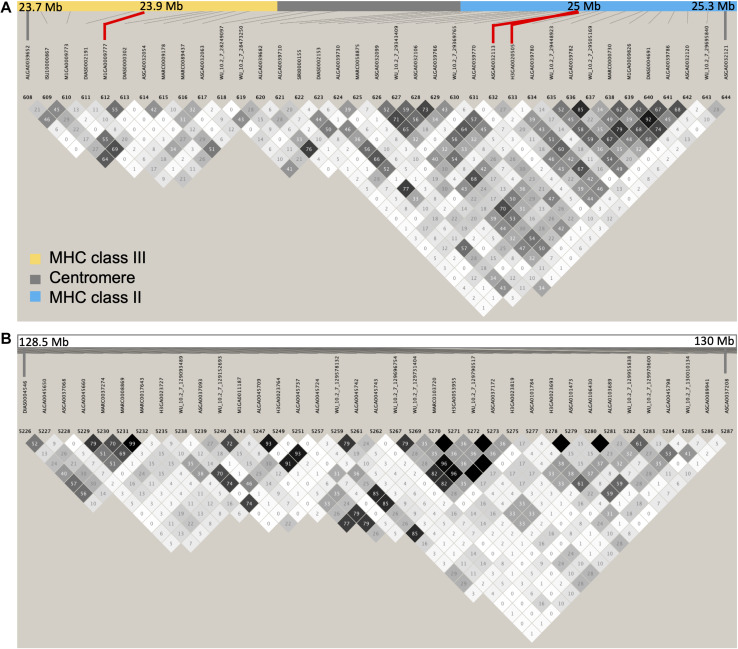
Linkage disequilibrium (LD) map. Plots represent LD map of the data used in our study for the **(A)** major histocompatibility complex (MHC) region (chromosome 7: 23–26 Mb) and **(B)** the quantitative trait locus (QTL) previously identified for sample-to-positive (S/P) ratio by Serão et al. (2014, 2016). The SNPs explaining greater proportion of the genetic variance for S/P ratio to porcine reproductive and respiratory syndrome virus vaccination (ASGA0032113, M1GA0009777, and H3GA0020505) and reproductive performance (H3GA0020505) are shown in red in panel **(A)**. The MHC regions class II (∼24.7–25.3 Mb) and class III (∼23.6–24.3 Mb) are in blue and yellow, respectively, separated by the centromere (∼24.3–24.7 Mb) being shown in gray. Differently than in Serão et al. (2014), our region on SSC 7 between 128.5 and 130 Mb (B) for their proposed QTL showed overall low LD.

Interestingly, in the bivariate GWAS, the major region associated with the covariance between S/P ratio and all reproductive performance was the MHC class II. Recently, a study with Landrace and Large White during a PRRS outbreak observed that sows carrying specific genotypes of the *TAP1* gene, located in this region, were more PRRS resilient than others (Laplana et al., 2020). In this study, female piglets at 6–7 weeks of age were PRRSV-vaccinated and, after a period, underwent a PRRS outbreak (Laplana et al., 2020). The resilience was measured as the capacity to maintain reproductive performance, such as NBA, during the outbreak (Laplana et al., 2020). Additionally, a polymorphism in the Hydroxysteroid 17-Beta Dehydrogenase 8 (*HSD17B8*) gene, located at 25.2 Mb, has been associated with reproductive traits in pigs (Ma et al., 2015). Haplotypes in the MHC class I and II regions have been previously associated with reproductive traits, such as ovulation rate, embryo development, and litter size in non-infected pigs (Vaiman et al., 1998). These results suggest that genes in the MHC region seem to have a direct effect on reproductive performance, and it is possible that these genes are in LD with genes controlling the immune response to PRRSV (Gautschi and Gaillard, 1990). Therefore, some of those candidate genes involved in the immune response to PRRSV and reproductive performance might be physically close to each other, which generates the high *r*_g_ between these two traits in our study and in Serão et al. (2014). On the other hand, the average LD within the MHC region (SSC 7: 23–26 Mb) was of *r*^2^ = 0.44, and the LD map shows very little overall LD in this region (Figure 5A), similarly to the one depicted by Serão et al. (2014), suggesting that the genetic correlation between S/P ratio and reproductive performance could be due to pleiotropy instead. The LD within a region may vary in different populations, which may explain, along with other environmental variations, the different results being obtained across the different studies. Finally, the MHC region was not identified in the univariate GWAS for reproductive performance (data not shown), nor any other major regions. Farrowing performance traits are explained by several loci with low effect along the genome; therefore, the use of bivariate analysis may be advantageous since measurements of one trait can be informative for other traits (Cheng et al., 2018a).

Interestingly, the other major QTL found by Serão et al. (2014, 2016) on SSC 7, called Mb 130, was not identified in our study. According to Serão et al. (2014), this region is in high LD and harbors genes associated with immune response, such as TNF receptor associated factor 3 (*TRAF3*), which is involved in the innate immune response and induces NF-kappa-B activation. We also constructed an LD map of this region (Figure 5B), but contrarily to Serão et al. (2014), we observed a low LD in this region. Hence, one possible explanation for the fact that we did not identify the region Mb 130 in our study may be due to SNPs on that region not being in LD with the QTL. In addition, although this region was identified in the overall GWAS in Serão et al. (2016), this region was not identified for all analyses when data on each one of the seven breeding companies were analyzed separately (NVL Serão, *personal communication*). Similarly, Hickmann et al. (2019) also did not find this region in multiparous Duroc and Landrace sows during a PRRS outbreak, using population of animals sourced from the same breeding company used in our study. Thus, another possible reason for not identifying this region may be that this region does not segregate in all commercial swine populations.

Summarizing, the major QTL identified in this study for vaccination was the same as the one previously identified in PRRSV-infected animals, supporting that the genetic control to vaccination may be similar to infection. This is reasonable, as we used an MLV vaccine, which has an attenuated form of the PRRSV. Also, the high genetic correlation between S/P ratio to PRRSV vaccination and reproductive performance, which was partially explained by this QTL, could be due to LD between genes controlling immune response and reproductive performance, or pleiotropy in the MHC region.

### Effect of Major Histocompatibility Complex Single-Nucleotide Polymorphisms on Antibody Response and Reproductive Traits

The main SNPs associated with S/P ratio were responsible for most of the variance accounted by the window, and after fitting them simultaneously as fixed effects in the model, the percentage of the variance explained by that window dropped to about null, supporting that these SNPs are playing a major role explaining the variability in S/P ratio. These three SNPs were not the same as the ones identified in Serão et al. (2014). Six out of 10 SNPs in the MHC region and in the Mb 130 region (unmapped in the current version of the SNP map) identified in Serão et al. (2014) were present in our dataset (positions based on the 11.1 assembly): ASGA003186 (SSC 7–22 Mb), MARC0058875 (SSC 7–24.8 Mb), ASGA0032151 (SSC 7–25.9 Mb), BGIS000074 (unmapped), MARC0037274 (unmapped), and ASGA0037093 (unmapped). We also evaluated their impact on S/P ratio by fitting all of them simultaneously as fixed effects in the model, along with the three SNPs identified in our analysis, but none of these six SNPs from Serão et al. (2014) were significantly (*P* ≥ 0.14) associated with S/P ratio in our dataset (data not shown).

The SNP H3GA0020505 was associated with S/P ratio and farrowing performance in the bivariate analysis. Although this was the SNP explained most of variance for all traits analyzed, after fitting it as a fixed effect in the statistical model, this SNP tended to be associated with NBA, MUM, and NSB. The AA genotype was favorable for S/P ratio, NBA, and NSB but not for MUM, being the heterozygous genotype (i.e., AB) the only simultaneously favorable for all these three traits. Assuming that greater S/P ratio is favorable, this SNP seems to have a complete dominance mode-of-action for the A allele, and the highest values of S/P ratio could be obtained with AA and AB genotypes. However, the AA genotype was unfavorable for MUM P3, with the other two genotypes (AB and BB) having the highest performance. For this trait, the dominance seems to be the opposite than for S/P ratio, with the B allele dominating the A allele. The biological explanation for this is beyond what can be concluded using SNP data, but in case of pleiotropy, the transcripts for of AB genotypes could be the only ones participating on complex pathways that favor both traits simultaneously. This same rationale could be used for PMW P2 and NSB P3, where AB and BB genotypes had similar and favorable performances, although no significant association between H3GA0020505 and these two traits were identified. The trend for these two traits was clear (i.e., AA > AB = BB) and similar to the that for MUM P3, but the lack of significant associations for PWM P2 and NSB P3 could be due to the high standard error of the AA genotype, which can be explained by its very low frequency in this population. The frequency of the AA, AB, and BB genotypes were 0.074, 0.464, and 0.462, respectively. The consistent superiority of the heterozygote across all of these traits facilitates selection for improved performance in this situation. Since these F1 gilts were generated from two maternal lines (Landrace and Large White), the selection for opposite alleles could be performed for each breed in order to result in 100% heterozygote F1 animals for this locus. Investigation of genotypic frequencies in the purebred populations used to create these F1 gilts indicates that this locus is in Hardy–Weinberg equilibrium (data not shown), suggesting that this novel locus has not yet been selected for.

We further explored the impact of H3GA0020505 on the relationship between S/P ratio and these traits discussed in this section. After fitting this SNP as fixed effect in the model, the covariance explained by the window containing this SNP dropped from 34 to 26% and 90 to 74% for NBA P1 and NSB PSY, respectively, but not for the other traits. The genetic correlation between these two traits and S/P ratio also decreased for NBA, from 0.61 to 0.50 for NBA P1, supporting the results that this SNP plays a major role in the relationship between these two traits. In addition, we observed that the genetic variances for S/P ratio and reproductive performance, and the genetic covariances between these traits decreased as well. Although this happened for all the traits, the genetic correlations between S/P ratio with PWM P2, NSB P3, and MUM P3 did not decrease (or had just a slight decrease; data not shown), showing that the remaining SNPs were capable of maintaining the relationship between these traits. Altogether, our results provided potential genetic markers (i.e., SNP) that could be explored in marker-assisted selection schemes to increase S/P ratio and farrowing performance in commercial sows.

### Genomic Prediction

The GPAs were moderate to high, showing that genomic information is able to accurately predict S/P ratio in commercial gilts vaccinated for PRRS. GPAs for ALL and MHC were similar and greater than for REST. Interestingly, results for REST using BayesC0 were greater than using BayesB. These results are in accordance with the GWAS results, in which the MHC region plays an important role in the prediction of S/P ratio and using only SNPs in the region promotes a substantial GPA. In contrast, no regions outside the MHC were identified in the GWAS, supporting the result using BayesC0 for REST, since this method assumes that no major QTLs control the trait being analyzed.

The use of the MHC region for selection should be taken with caution, since lower genetic variability in this region may be associated with limited immune response and, consequently, impaired productive/reproductive performance (Vaiman et al., 1998). If that is the case, it is valid to note that even after removing the MHC region for genomic prediction, the GPA was still moderate, indicating the possibility of not using the MHC region to promote genetic improvement for S/P ratio. Nonetheless, studies in the literature evaluating the impact of direct selection for changes in the swine MHC are scarce, and thus, additional studies are needed to better understand the negative impact that this selection may cause. Mallard et al. (1998) created lines of pigs with high and low immune response based on estimated breeding values for antibody and cellular immune response. Selection for high immune response improved the resistance to specific infectious pathogens and increased the weight gain without altering the *SLA-DRA* gene expression (Mallard et al., 1998). These results indicate that it is possible to select for lines based on immune response, and it would have an effect on the immune response to infectious diseases and productive performance. Interestingly, the heterozygote genotype of the SNP associated with antibody response and farrowing performance (i.e., H3GA0020505) had the most favorable performance for these traits, suggesting that selection of parents with opposite alleles may also be an alternative to improve these traits. Indeed, it has been previously shown that in humans and pigs, MHC compatibility in parents was associated with impaired pregnancy (Gautschi and Gaillard, 1990). This brings great possibilities for selection within line in the nucleus to obtain opposite homozygotes in maternal lines to create heterozygote F1 replacement gilts for improved reproduction at the commercial level. However, the impact of this selection on the performance of commercial three-cross hogs should also be evaluated to verify whether this locus has any impact on economically important traits in hogs.

Our results are slightly different from those reported by Serão et al. (2016). These authors performed analyses using phenotype and genotype data on replacement gilts to train markers and used two populations for validation: the purebred population under PRRS outbreak in Serão et al. (2014) and the same F1 population used to train markers in a seven-fold cross-validation. In Serão et al. (2016), GPAs using ALL were generally similar to using only markers in the MHC region, such as results found in our study. However, in our study, we found a much lower reduction in GPA for REST (0.50 ± 0.06) compared with ALL (0.52 ± 0.01) and MHC (0.55 ± 0.33), in contrast to in Serão et al. (2016), who reported a GPA of 0.12 for REST and 0.31 and 0.28 for ALL and MHC, respectively, when validating on crossbred animals. This must be due to the fact that in our study we used animals from the same breeding company, whereas in Serão et al. (2016), they used animals from different breeding companies. The greater genetic relatedness in our study should also be the main reason for overall greater GPAs compared with those in Serão et al. (2016). In fact, our results are more applicable to the industry than those of Serão et al. (2016), as selection is performed using animals from the same breeding company. Nonetheless, the use of vaccination versus infection could also help in explaining these differences. In general, these results indicate that the use of genomic information would be efficient to estimate genomic breeding values for S/P ratio.

## Conclusion

In this study, we showed that S/P ratio to PRRSV vaccination is moderately heritable and has favorable genetic correlation with reproductive performance in commercial pigs, especially with NBA at first parity, NSB and MUM in the third parity, and NSB per sow per year. A major QTL on the MHC classes II and III region explained most of the genetic variance of S/P ratio; and, more specifically, the region on MHC class II was associated with S/P ratio and reproductive performance, simultaneously. Three SNPs (ASGA0032113, H3GA0020505, and M1GA0009777) in these regions explained the vast majority of the genetic variance for S/P ratio within the MHC. In addition, SNP H3GA0020505 was associated with S/P ratio and reproductive performance, with the heterozygote genotype yielding the most favorable performance across these traits. Finally, the accuracy of genomic prediction was fairly high when using all SNPs available and only those located in the MHC region. Altogether, these results indicate that genetic selection for increased S/P ratio after PRRS MLV vaccination would result in indirect response for genetic improvement of farrowing performance in commercial sows. However, the improvement of reproductive performance may not be observed in all parities. Nonetheless, the majority of the genetic correlations that were not high were close to zero and/or in the right direction. Thus, genetic selection for S/P ratio might not generate unfavorable response in the reproductive performance in other parities. Future work is needed to validate our results for S/P ratio to PRRSV vaccination and its relationship with farrowing performance. In addition, it is necessary to evaluate if this genetic relationship exists in different populations (i.e., different breeding source), while evaluating additional time points for collection of S/P ratio and using data on sows with greater number of parities (i.e., >3).

## Data Availability Statement

The data that support the findings of this study are not publicly available. Data may be available from authors upon request and authorization from the company that generated the data.

## Ethics Statement

The animal study was reviewed and approved by Institutional Animal Care and Use Committee at Iowa State University (IACUC# 6-17-8551-S).

## Author Contributions

LS performed the data analyses, interpreted the results, and drafted the manuscript. KG, JD, MN, and NS developed the research project. LS, RF, JD, and NS conceived the statistical analyses. LS and NS prepared the first draft of the manuscript. KG and DL coordinated the collection of materials. MN and DL coordinated the antibody measurement analysis. All the authors contributed to the final manuscript, read and approved the final manuscript.

## Conflict of Interest

The authors declare that the research was conducted in the absence of any commercial or financial relationships that could be construed as a potential conflict of interest.
